# The CORE Group Polio Project’s Community Volunteers and Polio Eradication in Ethiopia: Self-Reports of Their Activities, Knowledge, and Contributions

**DOI:** 10.4269/ajtmh.18-1000

**Published:** 2019-10

**Authors:** Bethelehem Asegedew, Fasil Tessema, Henry B. Perry, Filimona Bisrat

**Affiliations:** 1 CORE Group Polio Project/Ethiopia, Addis Ababa, Ethiopia;; 2 Department of Epidemiology, Faculty of Public Health, Jimma University, Jimma, Ethiopia;; 3 Department of International Health, Johns Hopkins Bloomberg School of Public Health, Baltimore, Maryland

## Abstract

In 2001, the CORE Group Polio Project (CGPP) began to support polio eradication initiatives in hard-to-reach pastoralist and semi-pastoralist high-risk border areas of Ethiopia by training and supporting community volunteers (CVs) for immunization promotion and community-based surveillance activities. This article describes the development and current status of the CGPP CV network in Ethiopia. It also reports the results of a 2016 survey of CVs. Community volunteers are selected jointly by the local community, local government officials, and local health facility staff. They work closely with the health extension worker in their area and are responsible for 50–100 households. More than 12,000 CVs have been trained and have reached six million people. They make routine home visits to 1) provide education on vaccine-preventable diseases, 2) promote healthy behaviors, 3) inform parents on how to access immunization services, and 4) report cases of acute flaccid paralysis, neonatal tetanus, and measles as well as births. The 2016 survey of 675 CVs demonstrated that 84.1% had conducted home visits in the previous month to 1) identify and register pregnant mothers and newborns, 2) provide health education, 3) conduct disease surveillance, and 4) search for and register immunization defaulters. Of the CVs, 98.2% reported that their work had led to improvements in the community. Knowledge of CVs about vaccine-preventable diseases was suboptimal. CVs expressed a desire for more training. Community volunteers have made notable contributions to polio eradication efforts in high-risk areas of Ethiopia as well as to immunization promotion and disease control more broadly.

## INTRODUCTION

Community-based health volunteers represent an important resource for health programs throughout the world, particularly in resource-constrained settings.^1^ The 1978 Declaration of Alma-Ata, made at the International Conference on Primary Health Care at Alma-Ata, USSR (now Almaty, Kazakhstan), emphasized that 1) health problems cannot be solved without full participation and involvement of the community, 2) all community health strategies must address community needs at the local level, and 3) the development and implementation of these strategies must involve communities as stakeholders.^2^ Community-based health volunteers have been essential to the achievements of the Global Polio Eradication Initiative thus far. More than 10 million community-based health volunteers have supported supplemental immunization activities communities and administering oral polio vaccine.^3,4^

This article describes the development, knowledge, and practices of a specialized type of community volunteers (CVs) in Ethiopia who have been trained for a highly specific ongoing role in high-priority areas where polio immunization coverage is low, where the risk of active transmission of poliovirus is high, and where there is a need for stronger surveillance. The CORE Group Polio Project (CGPP) has trained and deployed CVs in sparsely populated regions of Ethiopia inhabited by pastoralist and semi-pastoralist populations and in border areas at high risk for transmission of poliovirus from neighboring countries. Included in this article are the findings of a 2016 survey of CVs. Another article in this series^5^ assesses the improvements in routine polio immunization coverage in the areas where these CGPP CVs have been active. A history of the CGPP’s global activities and its legacy are presented elsewhere in this series.^6,7^

## BACKGROUND OF THE CORE GROUP POLIO PROJECT CV PROGRAM IN ETHIOPIA

### The role of CVs in the CGPP implementation areas.

The CGPP has been working in Ethiopia since 2001 in pastoralist and semi-pastoralist areas and in high-risk border areas. In these areas, high levels of immunization coverage have been difficult to achieve. Wild poliovirus transmission persisted until 2014, when the last case of wild poliovirus was identified. The final cases of polio occurred in areas where levels of immunization coverage were low or where levels of immunization across the national border and the borders were porous and, therefore, where the risk of cross-border transmission was high.

The CGPP CVs are nominated by their respective communities. In addition, the lowest level of government administration (the *kebele,* or sub-district, with a population of approximately 5,000 people) and local health facilities are also involved in the selection of CGPP CVs. The criteria for selection of CGPP CVs include having interest in delivering health messages and health care to the local community, being well accepted by the community, being a good role model, speaking the local language, and living in the community.

Once selected, the CGPP CV begins to work with the health extension worker (HEW), a government paid, full-time community health worker (described further later). There are about three to five CGPP CVs per *kebele,* and each CV is responsible for surveillance of 50–100 households (and in some cases more).

The CGPP provides its CVs with 3 days of training on immunization, vaccine-preventable diseases, and community-based surveillance. The training is provided by field officers of non-governmental organizations (NGOs) that are CGPP project implementation partners as well as by HEWs. The CGPP implements its work through a secretariat, which engages international and local NGOs to take responsibility for CGPP activities in specific geographic areas.^6^ The training is based on a standardized training manual produced by the CGPP in Amharic and English as well as in local languages such as Somali and Oromifa. The training manual is distributed to all CVs and serves as a reference for all CVs, many of whom are illiterate. Literate family members assist illiterate CVs when necessary.

### The role and function of the CGPP CVs.

CVs are active in the areas where the CGPP is active. They exist in all 85 hard-to-reach pastoralist, semi-pastoralist, and international border areas of CGPP implementation districts of five regions of the country: Benishangul-Gumuz; Gambella; Oromia; Somali and Southern Nations, Nationalities, and Peoples (SNNP) Regions. CVs visit all households to provide education on vaccine-preventable diseases and on how to access immunization services. They also actively search for and report cases of the three types of diseases under surveillance: acute flaccid paralysis (AFP), measles, and tetanus. In addition, they identify newly pregnant women, newborns, and those in need of immunizations. The CGPP CVs provide oral or written reports to their supervising HEW, who then sends this information to the next higher level. The CGPP expects CVs to work 4 hours a week (2 hours per day for 2 days each week) at a minimum. CVs draft monthly plans and are supervised by HEWs to ensure that all households are reached within the 3-month reporting period. The order of visits is decided by the CVs. In addition, CVs make visits based on real-time information, such as new births in their communities or suspected AFP cases. They are supposed to visit four households per day (or eight households per week), spending half an hour with each household, and also to give priority to villages that are located farthest away.

### Supportive supervision.

Supportive supervision is a facilitative approach to supervision that promotes mentorship, joint problem-solving, and communication between supervisors and supervisees. Effective supportive supervision is critical for creating an enabling work environment, maintaining a well-functioning system, and motivating supervisees. Supportive supervision is provided for the CGPP CVs, both jointly and individually. Regular periodic joint supportive supervision using a standardized checklist is conducted by individual CGPP secretariat members, staff members from the CGPP Project NGO implementing partners, and the *woreda* (district) health office. Feedback from the supervision is given to supervisees and also to other stakeholders. Individual supervision is also provided by HEWs. CGPP/Ethiopia secretariat staff members conduct quarterly joint supportive supervision visits to his/her assigned areas during each supervision period, at which time they visit two *woredas*, one health center, two health posts, and three CGPP CVs in each *woreda*.

### Linkage of the CGPP CVs with the health system*.*

The CGPP CVs attend regular monthly meetings organized at the health post to which they are attached (and where two HEWs are based). At the time of these meetings, the CVs submit their monthly report, jointly review progress in the catchment area, prepare a work plan for the coming month, discuss constraints and solutions, and update their knowledge on surveillance and immunization. In addition, quarterly meetings are held at the *woreda* health office level where all the CGPP CVs, Health Development Army (HDA) volunteers (described later), HEWs, and *woreda* health officials meet. These meetings often have 100 participants. The monthly meetings take 1 day, and the quarterly meetings take 2–3 days. In addition to their regular meetings, CVs meet periodically with their supervising HEW, and together they exchange ideas and conduct house-to-house visits.

### Motivators and incentives for the CGPP CVs.

CVs are motivated by feelings of responsibility, trust, acceptance, and recognition by their communities to serve and promote immunization and health. Community volunteers are not paid but receive small non-financial incentives to motivate them. Together, the community, the CGPP secretariat, and the NGO partners provide a certificate of recognition for CVs, and the best performing CVs receive an award such as a mobile phone or radio. Other motivating influences include the supportive supervision and job aids provided by HEWs and *woreda* staff, the community’s acceptance of messages transmitted by the CGPP CVs, and opportunities to learn new and useful information.^8^ As an additional incentive, the CGPP provides its CVs with an umbrella, an apron, a bag, and a coat.

### Introduction of HEWs in 2003 and the HDA volunteers in 2012*.*

In the 1970s, community-based activities for maternal, child, and environmental health programs (including malaria control) in the Tigray regional state of northern Ethiopia began to use CVs.^9^ Similar activities spread throughout Ethiopia and led to the utilization of CVs by many NGOs and by vertical government programs such as family planning and HIV control.

In 2003, Ethiopia’s Federal Ministry of Health launched a new health-care plan, called the Accelerated Expansion of Primary Health Care Coverage, as part of a comprehensive Health Extension Program. The Health Extension Program represented an innovative, community-driven approach whose goal was the universal coverage of primary health care.^10,11^ These programs aimed to expand the health service coverage in all regions of Ethiopia, especially in rural parts of the country. The Health Extension Program is a comprehensive set of preventive, promotive, and curative services that have been combined as 17 separate packages, as shown in Table 1.

**Table 1:** The 17 packages of the Ethiopia Health Extension Program

Category	Packages
Disease prevention and control	1. TB, HIV/AIDS, and other sexually transmitted disease prevention and control
	2. Malaria prevention and control
	3. First aid and emergency measures
Family health services	4. Maternal and child health
	5. Family planning
	6. Immunization
	7. Adolescent reproductive health
	8. Nutrition
	9. Treatment of common childhood illnesses (diarrhea, pneumonia, malaria, and severe malnutrition)
Hygiene and environmental sanitation	10. Excreta disposal
	11. Solid and liquid waste disposal
	12. Water supply and safety measures
	13. Food hygiene and safety measures
	14. Healthy home environment
	15. Control of insects and rodents
	16. Personal hygiene
Health education and communication	17. Cross-cutting approaches

TB = tuberculosis.

Beginning in 2004, the Ethiopian government provided 1 year of training and deployed 38,000 full-time, salaried female HEWs to work at health posts, with two HEWs per health post and one health post serving a *kebele* (with approximately 5,000 people). In 2012, the government of Ethiopia introduced the HDA, which replaced most CV programs in health that had been developed by the government and NGOs for various purposes.^12^ Although the intention of government is to eventually replace all CVs with HDA volunteers, the CGPP CVs are still functioning in the CGPP implementation areas. In the future, many of the trained CGPP CVs will be absorbed into the HDA, and it is the government’s vision that the HDA will carry out polio eradication activities in the CGPP implementation areas where the HDA program is now not well-developed (namely in pastoralist, semi-pastoralist, and high-risk border areas). Trained informally by HEWs and working under their direct supervision, HDA volunteers provide health education and support social mobilization for health. They also serve as guides for HEWs when these workers come for community-level activities.

The HDA program is based on the identification and training of “model families” (who are exemplary in terms of health behaviors and health-care utilization). In practice, “model families” become leaders of a group of five families known as the “one-to-five network,” and these, in turn, combined to form a group of 25–30 “model households” within a village. Women who are living in “model households” become eligible to become an HDA volunteer after finishing 96 hours of training on the Health Extension Program and implementing model behaviors in their household.^12^ Nationwide, there are now approximately three million HDA volunteers.^12^ In the CGPP implementation areas where HDA volunteers are now active, the CGPP has trained HDA leaders (of which there is one for every 30 HDA volunteers) with the same training provided to CVs, and they take on the same tasks as CVs.

HDA volunteers collaborating with the CGPP take on the same responsibilities as CVs for polio eradication, promotion of routine immunization, and surveillance, as well as in supporting HEWs more generally in the implementation of the Health Extension Program. There is no literacy requirement to become an HDA volunteer. The informal training of HDA volunteers is facilitated by HEWs with support from primary health care units and *woreda* health offices. The training takes 15 days on average, and it focuses on high-impact maternal, newborn, and child health measures. In the training, in a *kebele* of 1,000 households (about 5,000 people), 150 HDA volunteers on average are expected to attend. After the introduction of the HDA, some of those who had been previously serving as a CGPP CV also became HDA volunteers. The HDA was intended to replace most other CV programs that had been developed by either the government or by NGOs before the introduction of the HDA in 2012.

### Differences between CGPP CVs and HDA volunteers.

The CGPP CVs are selected by the community together with the community leaders, the w*oreda*, the local *kebeles,* and HEWs. By contrast, the HDA has a tight governance structure; it is led by a command post established at each level of the government’s administrative structure: region, zone, district, and *kebele*. Just as for the CGPP CVs, there are no literacy requirements for HDA volunteers. However, in contrast to CGPP CVs, who are both men and women, all HDA volunteers, are women.

In areas where the CGPP is functioning, there is one CGPP CV for every 100–200 households, although there is an HDA volunteer for every five households, and the HDA volunteer is supposed to come from a model household. Whereas CGPP CVs work on immunization and on surveillance of vaccine-preventable diseases, HDA volunteers are responsible for promoting the 17 packages of the Health Extension Program (shown in Table 1). In addition, the HDA volunteers also promote development activities in education and agriculture. Although neither the CGPP CVs nor the HDA volunteers are paid, the CGPP CVs do receive incentives in the form of materials and certificates as well as “per-diem” payments for the days when they attend trainings.

### Recent activities and achievements of the CGPP CVs.

Since 2001, the CGPP has trained and placed more than 12,000 CVs along Ethiopia’s vulnerable borders with South Sudan, Kenya, and Somalia and in pastoralist/semi-pastoralist areas. As mentioned previously, the CGPP operates through a secretariat, hosted by the Consortium of Christian Relief and Development Associations, an Ethiopian NGO. The secretariat contracts with five international NGOs that contract with four national NGOs to implement the CGPP programs (Table 2). The CGPP CVs reach six million people in 85 *woredas*. At present, 11,650 CGPP CVs (many of whom are also HDA volunteers) make home visits, provide health promotion as described earlier, and look for cases of AFP, neonatal tetanus, and measles. In addition, they track the vaccination status of pregnant women and newborns.

**Table 2:** Current NGO partners of the CGPP/Ethiopia

International NGO partners	National NGO partners
Catholic Relief Services	Ethiopian Evangelical Church Mekane Yesus
Save the Children/Ethiopia	Ethiopian Orthodox Church
World Vision/Ethiopia	Pastoralist Concern
International Rescue Committee	Organization for Welfare Development in Action
African Medical and Research Foundation	–

NGO = nongovernmental organization.

During the 5-year period between 2012 and 2017, the CGPP CVs identified and referred 306,432 women in need of tetanus immunization, 181,192 newborns in need of immunization, and 71,904 other children younger than 1 year of age who had fallen behind on their immunization schedule.^13^ In addition to their achievements in expanding polio vaccine coverage and caregiver knowledge about polio which is documented elsewhere in this series,^5^ the CGPP CVs during this 5-year period were able to double the percentage of fully immunized children (from 25% to 44% according to data recorded on immunization cards), to increase the detection rate of non-polio AFP cases from 2.2 to 2.8 per 100,000 children younger than 15 years per year (compared with the national average of 2.5), and to maintain an adequacy of stool samples from children with AFP at 87–92%.^13^ These achievements are notable given that the areas where the CGPP operates are in the most challenging and hardest-to-reach areas of the country.

Having now described the CGPP CV program, we now proceed to describe the findings from a recent survey of the CGPP CVs in Ethiopia.

## METHODS

### Study population.

At the time of the 2016 survey, there were 11,650 CGPP CVs and HDA volunteers working in 85 districts with 1,638 *kebeles* in five regions of the country: Somali; Oromia; SNNPR; Benishangul-Gumuz; and Gambella.

### Study sample.

The survey consisted of 675 CVs of 2,857 CGPP CVs (excluding HDA volunteers) who were working in 11 zones†[Fn fn1] in four regions (Gambella, Oromia, Somali, and SNNPR) in intervention *kebeles* inhabited by pastoral and semi-pastoral people. Of the 723 *kebeles* in this geographic area, 223 *kebeles* were randomly selected. Within each of these selected *kebeles*, three CGPP CVs were randomly selected for interviews. One additional CV from each of six *kebeles* was interviewed, giving a total sample size 675.

### Data collection.

The study questionnaire was prepared in English and then translated into Somali, Oromifa, and Amharic languages. Face-to-face interviews were conducted in 2016 by data collectors who were community members with at least a high-school education and who were able to speak the local language in addition to Amharic. The data collectors received a 1-day training on the study objectives, selection of respondents, and interview procedures. The interviewers were supervised by the CGPP coordinators, temporary staff with experience in survey supervision, or government health officials.

### Data entry and management.

EpiData was used to enter the data with double entry verification. STATA version 13.0 (STATA Corp., College Station, TX) was used for data analysis.

### Ethical considerations.

The CGPP Ethiopia secretariat office provided a letter to the respective regional, zonal, and *woreda* health offices, and the *woreda* health officer provided a letter to each of the *kebele* offices requesting permission and collaboration in conducting the survey. Data collectors, supervisors, and coordinators were trained on the objectives of the study, interviewing techniques, research ethics, and data quality issues. Interviewers obtained consent from all respondents, and participation was voluntary. Verbal consent was obtained from each participant before beginning the interview. All participants were assured of the confidentiality of their responses. Because this survey was carried out as part of the ongoing monitoring and evaluation of the CGPP Ethiopia program, it was not considered to be research and was not submitted to an ethical review board for approval.

## RESULTS

### Sociodemographic characteristics of the respondents.

Of the 675 respondents, 51.5% (348/675) were female, 88.3% (596/675) were married, 58.9% were aged 30–49 years, 30.6% (207/675) were younger than 30 years, 10.2% were aged 50 years or older, 26.6% had no formal education, 51.9% (350/675) had 1–8 years of schooling, and 21.5% had attended grade 9 or above.

The distribution of CVs by region state was as follows: 45.6% were in Somali, 22.2% in Gambella, 19.1% (129/675) in Oromia, and 13.4% (90/675) in SNNPR. The regional state with the highest percentage of respondents who had no education was Somali (35.8%), whereas Oromia had the lowest (12.4%).

Of the respondents, 34.2% had resided in their *kebele* for more than 30 years, 58.5% had lived in the *kebele* for 10–29 years, and the rest (7.3%) had resided for less than 10 years. Of the respondents, 54.3% had served as a CGPP CV for 2–4 years, 32.3% had served in this role for five or more years, and 13.3% had served for fewer than 2 years. Of the respondents, 40.0% reported that they had been selected by the community and another 18.8% were selected by a committee composed of staff from the *woreda* health office, the primary health-care center, and the health post. The rest (41.1%) were selected by a combination of staff from health facilities, community leaders, and *kebele* administrators.

### Training received on vaccine-preventable diseases and AFP surveillance.

Among the 80.7% of the respondents who reported that they had received training provided by the CGPP, most (77.5%) received three or more days of training, whereas 12.4% reported fewer than 3 days of training and 14.2% received training for 7 days or more. Among the respondents with training, at least two-thirds had obtained training on the importance of immunizations, on registration of pregnant women and newborns, and on disease surveillance and reporting. Respondents reported that refresher training was given based on needs identified by supervisors. CVs sometimes leave abruptly and need to be replaced immediately. The replaced volunteers are sometimes not trained immediately and must be deployed without extensive training.

### CGPP/Ethiopia CVs’ knowledge about vaccine-preventable diseases.

**Figure 1.**
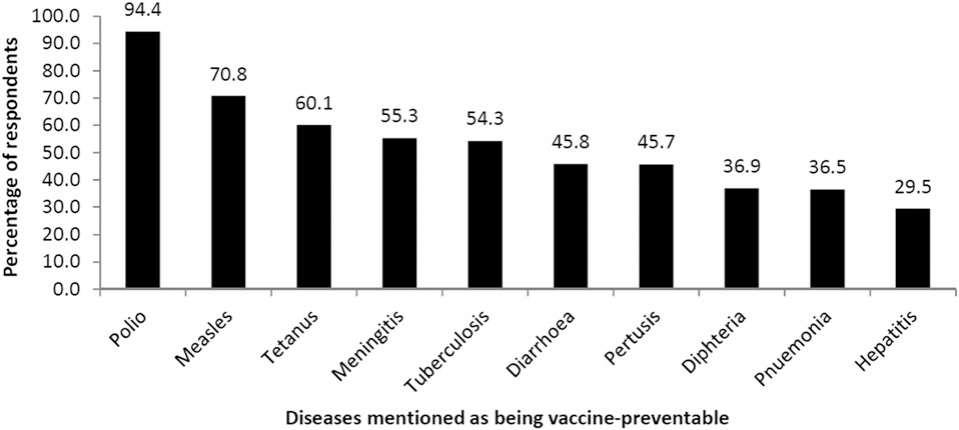
Percentage of the CGPP/Ethiopia CVs who spontaneously mentioned the diseases can be prevented by vaccination.

As shown in Figure 1, almost all respondents (94.4%) spontaneously knew that polio can be prevented by vaccination; 70.8% and 60.1%, respectively, were able to mention spontaneously that measles and tetanus can be prevented by vaccination. Between one-half and one-quarter of respondents mentioned spontaneously that diarrhea, pertussis, diphtheria, pneumonia, and hepatitis can be prevented by vaccination.

### Respondents’ knowledge about signs and symptoms of AFP.

Regarding the knowledge of CGPP CVs on signs and symptoms of AFP, 76.9% spontaneously mentioned limp limbs, 74.6% spontaneously mentioned cessation of walking or crawling as signs and symptoms of AFP, and 13.1% did not spontaneously mention any signs or symptoms. If a child with AFP was identified, 87.0% of CVs stated that they would accompany the parents and child to the nearest health facility, 34.4% said they would advise parents to do so, and 9.0% of CVs said that a child with AFP should be taken to a religious healer.

### Respondents’ activities during house-to-house visits.

Of the CGPP CVs, 84.1% reported that they had made home visits in the previous month. Among those who reported conducting home visits, 74.9% responded that they identified and registered pregnant mothers, 73.6% provided health education, and 58.3% identified and registered newborns. Of the respondents, 51.6% reported that they conducted disease surveillance (for polio, measles, and neonatal tetanus), 42.6% stated that they searched for and registered immunization defaulters, and 41.6% stated that they carried out social mobilization activities. Of the CVS, 84.2% reported that they delivered health education to mothers about immunization during the postpartum period, at which time they also provided education to the mother about newborn care.

### Linkage of CGPP CVs with the health system.

Of the respondents, 83.7% reported that they regularly attend monthly meetings at the health post. The reasons cited for not attending included that the meetings were not held (43.7%), the respondents were too busy (43.7%), or that the meeting was too far away from home (15.5%). Of the respondents, 89.1% said that they submitted monthly reports. The reports were submitted to health posts in a majority of the (73.8%) cases.

### Documentation of activities.

Of the respondents, 72.3% reported that they had a registration book in which they documented their activities. Among those who reported that they had a registration book, the presence of the book was confirmed by the interviewers in 73.1% of the cases. Of the registration books that were reviewed by the interviewer, 86.5% had a list of households that the CGPP CV had visited, 92.4% had a list of currently pregnant women, 82.3% had a list of children currently younger than 1 year, and 64.6% had a list of the children who needed immunization. Of the respondents, 74.4% confirmed that they still had the community-based surveillance training manual provided by the CGPP to use as a reference and 66.3% of respondents kept copies of the reports they had submitted. It should be noted that many CVs in remote pastoralist areas do not read or write. Therefore, these CVs submit their reports verbally to HEWs, who then wrote them down and sent them to the next higher level. These documents were not reviewed as part of the study.

### Supportive supervision for CGPP CVs.

Of the respondents, 68.0% reported that they had been supervised in the previous 3 months. Among those who had been supervised, one-half (54.0%) had been supervised by someone working for a CGPP NGO implementing partner and 46.2% by one of the *woreda* health office staff. Of the respondents, 30.1% said they had been supervised by a HEW, 21.2% by a staff member at the primary health-care center staff, 19.9% by a CGPP staff member (either a staff member of the secretariat office or of an NGO partner), and 32.0% had not received any supervision in the previous 3 months.

### CGPP CVs’ reports of their contributions to the Health Extension Program.

**Figure 2.**
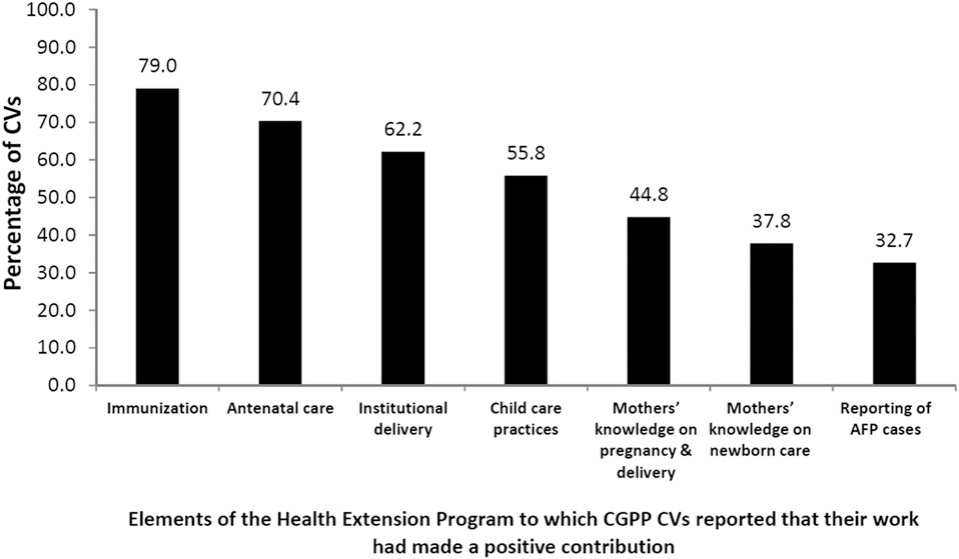
Opinions of the CGPP/Ethiopia CVs regarding what aspects of the Health Extension Program their work had contributed to.

CORE Group Polio Project CVs were asked about what contributions they thought they had made to the Health Extension Program. Almost all (98.2%) stated that their work had brought improvements. As shown in Figure 2, the majority (79.0%) stated that their work had led to improvements in immunization services, 70.4% reported that their work had led to improvements in antenatal care, and 62.2% reported contributions to improving institutional deliveries at the primary health-care center. One-third (32.7%) of the CVs indicated that they had contributed to the identification and reporting of AFP cases. This low percentage likely reflects the fact that only a small percentage of the CGPP CVs had actually identified a case of AFP.

Of the CGPP CVs, 72.3% of reported that they did not face any problems that hindered them from effectively accomplishing their activities. However, virtually all the respondents (94.9% or more) indicated that they would like more support such as more collaboration from community members; more training, information sharing, and supervision support from the HEWs; more collaboration from *kebele* administrators; and more support from primary health-care center staff and *woreda* health officers.

## DISCUSSION

The CGPP/Ethiopia has been a pioneer in the use of CVs for immunization promotion and surveillance in hard-to-reach and high-risk areas by training and supporting more than 11,000 CVs for supporting the polio eradication program and secondarily for promoting routine immunizations, disease surveillance (not only for polio but also for measles and tetanus), health education about pregnancy, delivery and newborn care, promotion of antenatal care at the health post, and promotion of delivery at the primary health-care center.

Our findings indicate that of 675 CVs interviewed, 81.1% reported that the community had participated in their selection. A majority of CVs had been living in their *kebele* for more than 10 years. Drawing CVs from the community and involving the community in their selection ensures a high level of trust between the CVs and the community. Although CGPP provides training and supportive supervision, 19.3% of CVs reported that they did not receive training before deployment. To ensure the success and capabilities of CVs, additional training is needed.

CVs are active and well linked to their supervising HEW and to the health system, and they feel that their efforts have been useful for a variety of important aspects of the Health Extension Program. CVs’ knowledge about vaccine-preventable diseases and AFP is reasonably good (94.4%), but only 70.8% and 60.1% knew that measles and tetanus can be prevented by immunization, indicating knowledge gaps. Evidence regarding the knowledge of CVs about immunizations might have been more favorable if the respondents had been prompted on these topics after being given the opportunity for spontaneous responses.

Most CVs conduct house-to-house visits to deliver immunization messages to mothers and caretakers, to register pregnant mothers and newborns, to identify those who are behind on their immunization schedule, and to notify the health facility of children needing immunizations.

Most CVs report that they are closely linked with their supervising HEWs and others in the health system who supervise them, and most of them report that they attend monthly meetings and submit monthly reports. However, 16% of respondents reported that they do not attend the monthly meeting because of lack of time or the long distance to the meeting venue. The activities of CVs are being documented well, and their activities reach down to every household for which they are responsible. They have a registration book of their activities that includes lists of households, pregnant women, children younger than 1 year, and immunization defaulters. However, illiteracy remains a barrier to documentation in some remote pastoralist areas. Illiterate CVs report orally to their supervising HEW who then write the information on to the monthly report form. Future studies should examine the utility of this process. They also keep the community-based surveillance training manual provided by the CGPP to use as a reference along with copies of the reports they submit to the health facility.

The contributions of CGPP CVs are not limited only to the activities mentioned in this article. They have also played an important role in improving polio immunization coverage in the CGPP catchment areas, as reported in a companion article in this series.^5^ Although there is a now-emerging, appropriate strong push globally to develop full-time, salaried Community Health Worker (CHW) cadres in low-income settings,^14^ the contributions of CGPP CVs documented in this article provide strong evidence that community-based volunteers can play an important role in addressing priority health problems, as have other recent reports.^1,15–20^

## CONCLUSION

Considering the situation of geographically inaccessible and hard-to-reach areas and the nomadic lifestyle of the population, the CGPP CVs have played an important role in promoting polio immunization and other basic health services. They have also contributed to the surveillance of vaccine-preventable diseases (polio, measles, and tetanus) and to increasing the knowledge of mothers about basic maternal and child health. Training, supportive supervision, and regular meetings have all improved the linkage of the CGPP CVs with the Health Extension Program and facilitated their contribution toward eradication of polio and control of other vaccine-preventable diseases. This strong performance of the CGPP CVs reflects a dedicated commitment from partners, including the local, national, and international NGOs who work with the CGPP, the government, and the polio eradication program in Ethiopia. Moving forward, community-based volunteers will be a valuable resource for addressing global, national, and local health priorities in resource-constrained settings.

The study also identified some areas where the CGPP CV program can be strengthened. Formal training of all CVs is necessary before field deployment. It is critical that attention be given to the elements that affect CV effectiveness throughout the implementation of the program. Training and supportive supervision are essential to maximize the effectiveness of CVs. The CGPP and other stakeholders need to carefully consider providing timely trainings before deployment, and supportive supervision must receive priority. Continued refresher training will also help build knowledge and capacity that will exist after CGPP is no longer operational. Training should continue to reinforce the understanding of polio vaccination and AFP detection while also increasing knowledge about other vaccine-preventable diseases.

## Footnotes

† † A zone is an administrative unit smaller than a region but larger than a *kebele*.

## References

[1] Leon N Sanders D Van Damme W Besada D Daviaud E Oliphant NP Berzal R Mason J Doherty T, 2015 The role of “hidden” community volunteers in community-based health service delivery platforms: examples from sub-Saharan Africa. Glob Health Action 8: 27214. 25770090 10.3402/gha.v8.27214 PMC4359271

[2] WHO, UNICEF, 1978 Declaration of Alma-Ata. International Conference on Primary Health Care, Alma-Ata, USSR, 6–12 September 1978. Available at: http://www.who.int/publications/almaata_declaration_en.pdf. Accessed April 22, 2019.

[3] Aylward RB Linkins J, 2005 Polio eradication: mobilizing and managing the human resources. Bull World Health Organ 83: 268–273. 15868017 PMC2626205

[4] Cochi SL Freeman A Guirguis S Jafari H Aylward B, 2014 Global polio eradication initiative: lessons learned and legacy. J Infect Dis 210 (Suppl 1): S540–S546. 25316878 10.1093/infdis/jiu345 PMC10544862

[5] Tessema F Bisrat F Kidane L Assres M Tadesse T Asegedew B, 2019 Improvements in polio vaccination status and knowledge about polio vaccination in the CORE Group Polio Project implementation areas in pastoralist and semi-pastoralist regions in Ethiopia. Am J Trop Med Hyg 101 (Suppl 4): 52–58. 10.4269/ajtmh.19-0022 PMC6776097 31760976

[6] Losey L 2019 The CORE Group Polio Project: an overview of its history and its contributions to the global polio eradication initiative. Am J Trop Med Hyg 101 (Suppl 4): 4–14. 10.4269/ajtmh.18-0916 PMC6776098 31760971

[7] Perry H Solomon R Bisrat F Hilmi L Stamidis K Steinglass R Weiss W Losey L Ogden E, 2019 Lessons learned from the CORE Group Polio Project and their relevance for other global health priorities. Am J Trop Med Hyg 101 (Suppl 4): 107–112. 10.4269/ajtmh.19-0036 PMC6776095 31760974

[8] Bilal N Herbst C Zhao C Soucat A Lemiere C, 2011 Health Extension Workers in Ethiopia: Improved Access and Coverage for the Rural Poor. Available at: http://siteresources.worldbank.org/AFRICAEXT/Resources/258643-1271798012256/Health_Extension_Workers_Ethiopia.pdf. Accessed April 19, 2019.

[9] Medhanyie A Spigt M Kifle Y Schaay N Sanders D Blanco R GeertJan D Berhane Y, 2012 The role of health extension workers in improving utilization of maternal health services in rural areas in Ethiopia: a cross sectional study. BMC Health Serv Res 12: 352. 23043288 10.1186/1472-6963-12-352 PMC3496586

[10] Wang H Tesfaye R Ramana G Chekagn C, 2016 Ethiopia Health Extension Program: An Institutionalized Community Approach for Universal Health Coverage 2016. Available at: https://openknowledge.worldbank.org/bitstream/handle/10986/24119/9781464808159.pdf?sequence=2&isAllowed=y. Accessed April 19, 2019.

[11] Assefa Y Tesfaye D Damme WV Hill PS, 2018 Effectiveness and sustainability of a diagonal investment approach to strengthen the primary health-care system in Ethiopia. Lancet 392: 1473–1481. 30343861 10.1016/S0140-6736(18)32215-3

[12] Perry H 2017 Case Studies of Large-Scale Community Health Worker Programs: Examples from Afghanistan, Bangladesh, Brazil, Ethiopia, India, Indonesia, Iran, Nepal, Niger, Pakistan, Rwanda, Zambia, and Zimbabwe. Available at: https://www.mcsprogram.org/resource/case-studies-large-scale-community-health-worker-programs-2/?_sfm_resource_topic=community-health. Accessed December 21, 2018.

[13] Stamidis K Bologna L Losey L, 2018 CORE Group Polio Project (CGPP) Final Evaluation Report 2017. Available at: https://coregroup.org/wp-content/uploads/2018/06/CGPP-Evaluation-Report-FINAL-5-10-2018.pdf. Accessed February 7, 2019.

[14] Cometto G 2018 WHO guideline on health policy and system support to optimize community health worker programmes. Lancet Glob Health 6: e1397–e1404. 30430994 10.1016/S2214-109X(18)30482-0

[15] Perry HB Sacks E Schleiff M Kumapley R Gupta S Rassekh BM Freeman PA, 2017 Comprehensive review of the evidence regarding the effectiveness of community-based primary health care in improving maternal, neonatal and child health: 6. Strategies used by effective projects. J Global Health 7: 010906. 10.7189/jogh.07.010906 PMC5491945 28685044

[16] Perry H Morrow M Davis T Borger S Weiss J DeCoster M Ricca J Ernst P 2015 Care groups II: a summary of the child survival outcomes achieved using volunteer community health workers in resource-constrained settings. Glob Health Sci Pract 3: 370–381. 26374799 10.9745/GHSP-D-15-00052 PMC4570012

[17] Neupane D McLachlan CS Mishra SR Olsen MH Perry HB Karki A Kallestrup P, 2018 Effectiveness of a lifestyle intervention led by female community health volunteers versus usual care in blood pressure reduction (COBIN): an open-label, cluster-randomised trial. Lancet Glob Health 6: e66–e73. 29241617 10.1016/S2214-109X(17)30411-4

[18] Tancred T Mandu R Hanson C Okuga M Manzi F Peterson S Schellenberg J Waiswa P Marchant T, EQUIP Study Team, 2018 How people-centred health systems can reach the grassroots: experiences implementing community-level quality improvement in rural Tanzania and Uganda. Health Policy Plan 33: e1–e13. 29304250 10.1093/heapol/czu070

[19] Maung CN Sein TT Hlaing T Okanurak K Silawan T Kaewkungwal J, 2017 Promoting community malaria control in rural Myanmar through an active community participation program using the participatory learning approach. Rural Remote Health 17: 4130. 28502184 10.22605/RRH4130

[20] Fredricks K Dinh H Kusi M Yogal C Karmacharya BM Burke TF Nelson BD, 2017 Community health workers and disasters: lessons learned from the 2015 earthquake in Nepal. Prehosp Disaster Med 32: 604–609. 28786371 10.1017/S1049023X1700680X

